# Factors associated with humeral shaft fracture nonunion

**DOI:** 10.1007/s00590-026-04666-5

**Published:** 2026-02-02

**Authors:** Ellen Lutnick, Bradley Hawayek, Marco Flores, Kevin Schauer, Elias Joseph, Mohammad Haider, Matthew Binkley

**Affiliations:** 1 University at Buffalo, State University of New York, Buffalo, USA; 2 West Virginia University, Morgantown, USA

**Keywords:** Humeral shaft fracture, Humeral fracture nonunion, Nonunion, Humeral shaft ORIF

## Abstract

**Purpose:**

This study aims to describe factors associated with risk of nonunion in patients treated for humeral shaft fracture.

**Methods:**

Adult patients treated for humeral shaft fracture at one Level 1 center from December 2009-July 2020, screened via ICD-10 code were retrospectively reviewed. Patient characteristics and radiographic analysis were recorded. Independent samples t-test was used for continuous variables, and chi squared or fishers exact test for categorical. *P* < 0.05 was considered significant.

**Results:**

Of 386 patients, 15.3% went onto nonunion (mean time to diagnosis 122 days). Patients with nonunion were more likely have comorbid alcoholism (*p* < 0.001) and hypothyroidism (*p* 0.048) and were significantly more likely to have been initially managed nonoperatively (*p* < 0.001). Mechanism was significantly associated (*p* 0.008). Of those with nonunion treated surgically initially, there were differences in the number and positioning of screws utilized in ORIF, including more screws distally (4.29+/− 1.86 vs. 3.77+/− 0.92, *p* 0.048) to the fracture through the plate, and fewer screws outside of the plate in the form of lag screws (0.41+/− 0.87 vs. 1.13+/− 1.44, *p* 0.045) in diagnosed nonunion. 11 patients diagnosed with nonunion required more than 1 revision procedure (mean 2.18). 27 patients diagnosed with nonunion had available documented radiographic union (range 56–872 days from date of nonunion diagnosis).

**Conclusion:**

Specific comorbidities, initial nonoperative management and surgical construct configuration in patients treated with ORIF are risk factors for development of humeral shaft fracture nonunion. Treatment and recovery from humeral shaft nonunion remain varied, warranting further study.

## Introduction

 Humeral shaft fractures account for an estimated 1–3% of all fractures [1]. Traditionally, most fractures have been managed non-operatively with high union rates with functional bracing developed by Sarmiento [2]. However, despite high union rates of nonoperative treatment, several studies have demonstrated that union rates are better with surgical intervention [3, 4]. Indications for surgical intervention include open fracture, polytrauma, vascular injury, and failed nonoperative treatment [5]. The main operative techniques described in the literature include open reduction and internal fixation (ORIF), intramedullary nail fixation (IMN) and minimally invasive percutaneous osteosynthesis (MIPO) [5]. Each have been described in literature to have satisfactory union rates ranging from 84 to 97% [6]. Despite this, nonunion continues to be a concern in these fractures with nonunion rates ranging from 2 to 33% [7, 8]. 

Often, the cause of fracture nonunion is multifactorial [9]. Transverse displaced fractures and middle to distal third fractures of the humerus have been reported to confer higher rates of nonunion [10]. Older age, infection, and comorbidities such as diabetes and osteoporosis have been described as contributors [9]. Furthermore, modifiable factors such as smoking, non-steroidal anti-inflammatory medication use, corticosteroid use, and poor patient compliance have been reported to contribute to worse outcomes and higher nonunion rates [11]. 

Currently, debate exists with consideration for when to appropriately directly intervene with surgical management without an initial trial of nonoperative treatment. There exist many considerations for surgical vs. non-operative management for the treatment of humeral shaft fractures, including injury characteristics and patient factors. Despite its potential advantage, surgical risk must always be considered. The purpose of this retrospective cohort study is to compare the rates of nonunion in patients initially treated nonoperatively and operatively for humeral shaft fracture, and to determine what risk factors are associated with nonunion.

## Methods

### Study design

This study is a single center, retrospective cohort analysis conducted at one academic Level 1 Trauma Center. The study was designed to evaluate the outcomes of treatment for humeral shaft fracture, including incidence and risk factors for nonunion in patients treated between December 1, 2000, and July 31, 2020.

### Inclusion and exclusion criteria

Inclusion criteria encompassed all adult patients (18 years and older) treated for humeral shaft fracture, identified using the AO/OTA classification (12) and corresponding International Classification of Disease (ICD-10) codes (i.e. S42.3X), at our institution within the specified time frame. AO/OTA classification was verified by orthopaedic surgery residents contributing to a Fracture Registry maintained by the institution. Patients were excluded if they had humeral fractures other than humeral shaft fractures, including those extending into the proximal or distal humerus as defined by AO/OTA classification, underwent definitive treatment at another facility, had initial admission outside the study period, or had incomplete records with regards to accessible radiographs at time of injury.

### Patient grouping

Patients were categorized into two groups based on the method of initial treatment as operative vs. non-operative. Treatment by either methodology was decided at the discretion of the treating surgeon. Nonoperatively treated humeral shaft fractures at our institution were conventionally immobilized with plaster splinting, followed by transition to Sarmiento bracing in an outpatient setting at approximately 1–2 weeks from the time of initial injury and splint placement. Occasionally, placement of a Sarmiento brace in the Emergency Department setting was performed based on brace availability and various patient factors.

All patients who were initially treated with surgical intervention were temporized until the date of surgery with a plaster coaptation splint. Patients were treated via a variety of surgical techniques and approaches, including open reduction internal fixation with a plate and screws (ORIF), intramedullary nailing (IMN), or minimally invasive percutaneous osteosynthesis (MIPO). The type and positioning of hardware, including the number of screws both within a plate construct or independent of a plate were chosen at the discretion of the treating surgeon, with consideration for patient anatomy, fracture morphology.

### Data collection

Data were extracted from the electronic medical records of the participating institution using a standardized chart review protocol. The data collected included demographic information, clinical and surgical characteristics, and outcomes including nonunion. Specific variables included patient age, sex, body mass index (BMI), smoking status, comorbidities, mechanism of injury, and fracture classification according to the AO/OTA system. Surgical details such as method of surgical fixation, perioperative complications, and return to the operating room were meticulously extracted from the record. Mechanisms of injury was defined as high energy vs. low energy, with high energy including any motorcycle accidents, motor vehicle accidents, or falls from > 4 feet. Ground level falls were considered low energy.

### Outcome measures

The primary outcome was the incidence of nonunion. Nonunion was defined as either clinical evidence of persistent motion across the fracture site at 6 weeks and/or lack of evidence of bridging callus at 3 months on radiographic analysis without evidence of progressive bone healing. These findings were corroborated by electronic medical record retrospective review, in which the treating surgeon documented formally that they felt the patient’s radiographs and clinical exam necessitated further treatment for nonunion [8, 12, 13]. 

Typically, patients with these signs/symptoms present were followed more closely, and specific mention of formal nonunion diagnosis in the electronic medical records on retrospective review correlates to the time to nonunion diagnosis, often after clinical concern was raised based on clinical/radiographic findings. Secondary outcomes included factors predictive of nonunion in the overall cohort, including patient demographics/comorbidities, and radiographic analysis including the quality of reduction, measured as any remaining gap at the site of fracture after initial treatment via surgical vs. non-operative management, and description of the surgical hardware utilized in the setting of operative intervention. Radiographic analysis was performed by three senior orthopedic surgical residents, and any discrepancies were reviewed and decided upon by the senior author (MB).

### Statistic analysis

Patient characteristics and radiographic analysis were recorded. Descriptive statistics (mean and standard deviation for continuous variables, frequencies, and percentages for categorical variables) were calculated for the entire cohort and stratified by union vs. nonunion. Independent samples t-test was used for continuous variables, and chi squared or fishers exact test for categorical. *P* < 0.05 was considered significant. All analyses were performed using SPSS.

### Ethical considerations

The study was approved by the Institutional Review Boards (IRB) of the participating institution. All data collection and storage complied with HIPAA regulations, and all members of the research team were trained in data security and patient confidentiality.

## Results

386 patients were included for analysis. 15.3% of patients (n 59) went onto nonunion (mean time to diagnosis 122 days). Table 1 describes the demographics of the included patients.

**Table 1:** Union vs. Nonunion demographics

	Union:	Nonunion:	*P* value:
N	327 (84.7%)	59 (15.3%)	
Age (years)	56.21+/− 22	59.83+/− 17.71	0.233
Sex			0.264
Female	152 (46.3%)	32 (54.2%)	
Male	176 (53.7%)	27 (45.8%)	
BMI	29.26+/− 7.81	29.54+/− 8.5	0.819
Smoking			0.582
Current	76 (23.2%)	1 (30.5%)	
Former	37 (11.3%)	7 (11.9%)	
Never	178 (54.3%)	30 (50.8%)	
Occupation			0.699
Employed	76 (23.2%)	15 (25.4%)	
Unemployed	82 (25.0%)	21 (35.6%)	
IDDM	23 (7.0%)	3 (5.1%)	0.573
NIDDM	22 (6.7%)	5 (8.5%)	0.644
Dialysis	3 (0.9%)	0 (0%)	0.458
Alcoholism	10 (3.0%)	8 (13.6%)	**< 0.001**
Osteoporosis	11 (3.4%)	1 (1.7%)	0.425
Hypothyroidism	36 (11.0%)	12 (20.3%)	**0.048**
ASA			0.37
1	16 (4.9%)	2 (3.4%)	
2	100 (30.5%)	22 (37.3%)	
3	65 (19.8%)	21 (35.6%)	
4	6 (1.8%)	0 (0%)	
5	2 (0.6%)	0 (0%)	
Mechanism			**0.008**
Low energy	146 (44.5%)	38 (65.5%)	
High energy	171 (52.1%)	20 (34.5%)	
GSW	11 (3.4%)	0 (0%)	
Additional Fractures			0.207
Ipsilateral upper extremity	20 (6.1%)	1 (1.7%)	
Contralateral upper extremity	14 (4.2%)	3 (5.1%)	
Lower extremity/pelvis	46 (14%)	8 (13.6%)	
Spine	15 (4.5%)	4 (8.5%)	
Open Fracture	35 (10.7%)	6 (10.2%)	0.908
Associated injury			0.191
None	297 (91.1%)	55 (93.2%)	
Abdominal injury	13 (4.0%)	1 (1.7%)	
Chest injury	12 (3.1%)	2 (3.4%)	
Head injury	9 (2.7%)	1 (1.7%)	

Bolded text denotes statistical significance considering p < 0.05

Patients with nonunion were more likely have comorbid alcoholism (*p* < 0.001) and hypothyroidism (0.048). Smoking status was not significantly associated with nonunion risk (*p* 0.582). Neither insulin dependent (IDDM) nor noninsulin dependent (NIDDM) diabetes were significantly associated with nonunion. Osteoporosis was also not found to be significant on comparison; however, only 12 patients in the entire cohort had documented diagnoses of osteoporosis.

Mechanism was significantly associated with nonunion. Patients with nonunion had significantly higher proportion of injuries due to a low-energy mechanism than patients who went on to union after initial treatment (65.5 vs. 44.5%; *p* 0.008).

Patients with nonunion were significantly more likely to have been initially managed nonoperatively (*p* < 0.001; Table 2). Patients treated surgically were predominately treated via ORIF (n 193). Surgical technique and approach were not found to be significantly associated with nonunion; however, there were only 9 patients treated with IMN and 4 treated with MIPO. Fracture location as in the proximal, middle, or distal 1/3rd humeral shaft was not associated with nonunion.

**Table 2:** Union vs. Nonunion treatment data

	Union:	Nonunion:	*P* value:
Treatment			**< 0.001**
Operative	193 (58.8%)	17 (28.8%)	
Nonoperative	135 (41.2%)	42 (71.2%)	
Type of surgery			0.724
ORIF	193 (58.8%)	17 (28.8%)	
IMN	9 (2.7%)	0	
MIPO	4 (1.2%)	0	
RSA w/ long stem	1 (0.3%)	0	
Approach			0.227
Anterior	107 (32.6%)	12 (20.3%)	
Posterior	86 (26.2%)	5 (8.5%)	
AO Classification			–
11A2	1 (0.3%)	0	
12A1	73 (22.3%)	12 (20.3%)	
12A2	44 (13.4%)	6 (10.2%)	
12A3	81 (24.7%)	13 (22%)	
12B1	26 (7.9%)	4 (6.8%)	
12B2	54 (16.5%)	13 (22%)	
12B3	13 (4.0%)	3 (5.1%)	
12C1	3 (0.9%)	1 (1.7%)	
12C2	12 (3.7%)	2 (3.4%)	
12C3	15 (4.6%)	4 (6.8%)	
13A2	1 (0.3%)	0.00%	
13A3	2 (0.6%)	1 (1.7%)	
Fracture location:			0.289
Proximal	69 (21.2%)	15 (25.4%)	
Middle	156 (47.9%)	34 (57.6%)	
Distal	98 (30.1%)	10 (16.9%)	
Complications:			–
Radial nerve injury after injury	40 (12.5%)	10 (17%)	
Radial nerve injury after surgery	1 (0.3%)	1 (1.7%)	
Infection with surgery	5 (1.5%)	2 (3.4%)	
Infection without surgery	2 (0.6%)	0 (0%)	
Death	2 (0.6%)	0 (0%)	
Malunion	5 (1.5%)	0 (0%)	
Adhesive capsulitis	1 (0.3%)	0 (0%)	
Compartment Syndrome	1 (0.3%)	0 (0%)	
Subsequent fracture/hardware failure	8 (2.4%)	7 (11.9%)	
Elbow contracture	1 (0.3%)	0 (0%)	
Time to last follow up (days)	206.49+/− 316.82	414.53+/− 395.46	–

Bolded text denotes statistical significance considering p < 0.05

**Table 3:** Hardware utilized in ORIF and risk of nonunion

	Nonunion (*n* = 17) Mean ± SD	Union (*n* = 182) Mean ± SD	*p*-value
Screws proximal to the fracture through the plate	4.35 ± 1.320	3.84 ± 1.042	0.06
Screws distal to the fracture through the plate	4.29 ± 1.863	3.77 ± 0.922	**0.048**
Screws through plate in fracture	0/35 ± 0.996	0.61 ± 1.126	0.365
Screws outside plate	0.41 ± 0.870	1.13 ± 1.443	**0.045**

Bolded text denotes statistical significance considering p < 0.05

Of those with nonunion who were initially treated surgically, there were differences in the number and positioning of screws utilized in ORIF, including more screws distally (4.29+/− 1.86 vs. 3.77+/− 0.92, *p* 0.048) to the fracture, and fewer screws outside of the plate in the form of lag screws (0.41+/− 0.87 vs. 1.13+/− 1.44, *p* 0.045) in diagnosed nonunion (Table 3). Of 59 patients diagnosed with nonunion, 11 required more than 1 revision procedure (mean 2.18).

Amongst those complications reported, post-operative radial nerve palsy was higher in the non-union group compared to the group that had successful union after initial treatment; however, this number included radial nerve palsies which occurred after subsequent operations to treat the nonunion. Infection after surgical management nearly doubled in those who were diagnosed with nonunion compared to those who went onto union after initial management (3.4% vs. 1.5%). Subsequent refracture or hardware failure rates were also higher amongst those diagnosed with nonunion after initial treatment (11.9% vs. 2.4%). Rates of follow up were longer in those diagnosed with nonunion (Table 2); however, only 27 of 59 patients diagnosed with nonunion had available documented radiographic union (range 56–872 days from date of nonunion diagnosis).

## Discussion

Humeral shaft fracture nonunion rates vary in the literature with reports ranging from 2 to 33% [7, 8]. In the meta-analysis done by Lode at al., nonunion rates with nonoperative treatment ranged from about 0–27% and operative nonunion rates ranged from 0 to 19% [14]. In our study, 59/386 patients (15.3%) experienced nonunion which is consistent with previous literature. Multiple risk factors for nonunion after humeral shaft fracture have been previously reported. Olson et al. demonstrated tobacco smoking, alcohol abuse and cirrhosis all to be independent risk factors for nonunion [6]. Our analysis revealed alcoholism and hypothyroidism to be significantly associated with nonunion. This supports previous studies that cite patient comorbidities contributing to metabolic abnormalities and malnutrition as risk factors for nonunion. In addition, we also identified low-energy mechanism to be significantly associated with risk of nonunion. These low-energy mechanisms may be associated with advanced patient age, and subsequently increased incidence of osteoporosis [9], a known risk factor for nonunion in the setting of humeral shaft fracture. Although we did not identify osteoporosis as a risk factor for nonunion within our own cohort, it is possible that patients who sustained injuries from low-energy mechanisms had poorer bone quality. Despite lack of formal diagnosis of osteoporosis, this potentially supports the hypothesis that poor bone quality and osteoporosis are risk factors for nonunion.

Fracture location and pattern have been additionally demonstrated as associated with nonunion. Neuhaus et al. demonstrated a higher risk of nonunion in proximal and midshaft humeral fractures compared to fractures of the distal shaft [15]. Although the results of our study did not reach statistical significance, there was a trend toward a higher percentage of proximal and middle-third humeral shaft fractures amongst those that went onto nonunion after initial treatment, and a higher percentage of distal fractures in the union group. In the same study, 20% of the patients with transverse or short oblique fractures of the mid-diaphyseal portion of the humerus (AO types 12A2 or 12A3) underwent operative intervention following at least 6 weeks of treatment with immobilization due to concern for persistent motion across the fracture site. Our study did not identify a significant association between AO fracture type and risk of nonunion; however, this may be secondary to the retrospective nature of the study. Patients with transverse or short-oblique fractures of the mid-diaphyseal region of the humerus may have been more likely to be initially managed operatively, which may have resulted in fewer nonunions in this subgroup of patients.

Initial nonoperative management has also been shown to be a risk factor for nonunion. Traditionally, nonoperative management with functional bracing of humeral shaft fractures has demonstrated good results, including a union rate as high as 97% [2]. More recently, several studies have demonstrated higher union rates with operative treatment. Van de Wall et al. demonstrated conservative treatment of humeral shaft fractures to be associated with higher nonunion rate when compared to operative management [3]. In their systematic review and meta-analysis, nonunion occurred in 15.3% of patients treated nonoperatively compared to 6.4% in those treated operatively. Reintervention was also higher in patients who were initially treated nonoperatively, most commonly in the form of delayed operative intervention due to nonunion. Retrospective reviews by Harkin et al. and Denard et al. separately analyzed results from 126 to 213 patients respectively, with both studies concluding higher union rates with operative intervention compared to conservative management (33% vs. 4% nonunion in conservative vs. operative management, and 20.6% vs. 8.7% nonunion in conservative vs. operative management, respectively) [4]. ^,^ [16] Among one randomized control trial comparing functional bracing and ORIF [17], a 6.6% increase in the risk of nonunion, diagnosed at 6 months after injury, was demonstrated for fractures treated with functional bracing compared to those treated by ORIF, although this difference did not reach statistical significance, likely due in part to the relatively small cohort described (sample size 60 patients). Our study demonstrated that a significantly higher percentage of patients who were initially managed nonoperatively went on to nonunion compared to those initially managed operatively. This data supports a growing consensus that operative treatment may lead to better outcomes with quicker return to mobility and function and lower rates of nonunion [13, 18, 19]. Considering cost analysis, operative management may provide a cost-effective option when considering the cost of lost wages in a slower return to function in more recent analyses [20, 21]. Kumar et al. and Matsunaga et al. both support this with their patients who were treated operatively reporting significantly better DASH scores compared to nonoperative treated patients [18, 22]. Schoch et al. further supports this consensus with perceived return to function due to brace removal occurring on average of 11.5 weeks with nonoperative management compared to 6.3–9.8 weeks with intramedullary nailing and 8.9–10.4 weeks with compression plating [23]. Among patients initially treated with operative intervention for humeral shaft fractures, method of fixation has been shown to have variable results regarding union rates. Kurup et al. performed a systematic review of multiple randomized control trials comparing dynamic compression plating and locked IMN for humeral shaft fractures. Their findings noted increased frequency of shoulder impingement with intramedullary nailing [24]. However, there was no difference in fracture union rates. Meta-analyses performed by Ouyang et al. and Dai et al. also demonstrated no significance difference in nonunion with plating compared to IMN fixation [25, 26]. Studies examining the use of minimally invasive plating osteosynthesis (MIPO) have described higher union rates compared to other operative interventions, 97% with MIPO compared to 94.5% for ORIF [27]. These findings have been attributed to advantages of MIPO including less devitalization of the bone from soft-tissue stripping, resulting in vascular preservation. However, studies examining larger sample sizes via prospective design are required to truly determine if this difference is significant. Tetsworth et al. also reported on the advantages of MIPO compared to IMN as having fewer complications and faster time to radiographic union while also being less invasive per MIPO technical definition [28]. Additionally, MIPO has been described as having a physiologic advantage encouraging healing with minimal disruption of the surrounding soft tissue envelope in the setting of comminuted fractures [29]. In our study, 93.75% of patients who were initially managed operatively underwent ORIF. Nine patients underwent IMN fixation, and 4 patients underwent MIPO; there were no cases of nonunion described in those patients treated initially with IMN or MIPO. However, these groups were underpowered to compare union rates across fixation strategies.

Of those with nonunion who were initially treated surgically with ORIF, there were differences in the number and positioning of screws utilized, including more screws distal to the fracture through the plate, and fewer screws utilized outside of the plate. This appears to be reflective of differences in fixation principles in the use of ORIF may impact union rates. Specifically, more screws outside of the plate in the form of lag-screw fixation suggests that fracture patterns amenable to lag-technique and neutralization plating may have a lower risk of non-union compared to other fixation techniques. This may be partially a result of the relative simplicity of the fracture pattern, or more likely a result of the quality of reduction required by this treatment strategy, including meticulous surgical technique required for fracture reduction and compression across the fracture required for lag screw utilization. The use of more screws distal to the fracture through the utilized hardware may suggest the risk of nonunion is higher when utilizing bridge-plating technique with a shorter relative working length of the construct utilized. Ultimately, adhering to standard AO principles when using ORIF for treatment of humeral shaft fractures is important, including consideration for fixation with lag-technique, or appropriate bridge-plating when the fracture orientation is deemed amenable to these types of fixation.

The underlying etiology of humeral shaft nonunion has been largely attributed with inadequate internal fixation and underlying patient physiology, especially metabolic comorbidities [30, 31]. Treatment of nonunion typically requires a more extensive dissection and debridement, special consideration of contributing patient factors, including nutritional status and the potential of infection, as well as specific surgical approach and technique that allows for the nonunion to be adequately addressed, typically in the form of open reduction and internal fixation with the use of bone grafting [32]. Of those patients within our cohort, 59 were ultimately diagnosed with nonunion at an average of 4 months from initial treatment. 11 of these patients required more than 1 revision procedure (mean number of procedures 2.18), representing nearly 19% of patients treated surgically for nonunion. Additionally, the risk of post-surgical infection more than doubled amongst those patients ultimately treated surgically for nonunion, demonstrating the patient morbidity associated with nonunion in this setting. Twenty-seven patients had available documented radiographic union ranging from 56 to 872 days from date of diagnosis. This represents a substantial burden of morbidity to the patient, as well as additional financial burdens associated with treatment costs as well as a wide range of potential time lost to disability.

While our average time to diagnosis of nonunion at about 4 months may at first glance appear premature to traditional definitions requiring 6 months of follow up, it is commonly accepted that gross motion at the fracture site in patients treated nonoperatively at 6 weeks is highly suspicious for nonunion, and lack of healing progression, without any bridging callous by 3 months are included amongst acceptable definitions. More importantly, surgeon discretion, including requirement for decisions for further treatment related to diagnosed nonunion were used to confirm the diagnoses in this analysis, in addition to careful independent radiographic analysis and chart review for documentation of continued motion at the fracture. Similar definitions have been previously reported amongst published literature, including consideration for delayed operative treatment in the setting of failure of significant healing with conservative management [12]. Amongst other similar previously published cohorts, while there exists overlap in diagnostic criteria, the actual time to nonunion diagnosis is commonly not reported [8], a limitation of any definition, as treatment for nonunion in the clinical setting may occur before a set time point depending on patient symptoms and shared decision making. In the FISH Randomized Clinical trial, nonunion was defined as “no bridging fracture callus in 3 of the 4 cortices in anteroposterior and lateral radiographs taken at 3 months or later after the fracture and a notion of clinically verified motion at the fracture site,” [13] which is how nonunion and radiographic analysis was defined in this study.

This study has several limitations. The retrospective nature of the study presents the possibility of selection bias related to patient selection for initial treatment via operative vs. nonoperative management. Additionally, alternative previously published definitions of humeral shaft nonunion, including specific endpoints as far from initial injury as 4–6 months, were unable to be explored as primary endpoints in this analysis, as the definition of nonunion described in these methods were the definition utilized to guide clinical management in this cohort. Identification of patients via ICD-10 code may have resulted in the inappropriate exclusion of patients with inaccurate diagnostic codes not able to be identified for review. Also, the small number of patients treated initially with IMN and MIPO inhibits our ability to compare union rates among initial surgical fixation strategies; the subgroup analysis is underpowered to draw definitive clinical conclusions with consideration for implant configuration. Similarly, rates of specific complications were low such that analysis examining for any associations to other clinical factors were underpowered to meaningfully determine significance. Final radiographic follow up was limited amongst our cohort of patients diagnosed with nonunion, and amongst those with available radiographs, the time to union was documented over a range of 56–872 days. This attrition and extreme variability limit the ability to draw conclusion from presented analysis with regards to treatment and recovery from nonunion. Lastly, while our study specifically sought to identify nonunion rates and risk factors for nonunion in patients with humeral shaft fractures, functional outcomes were unavailable for most patients, limiting our ability to determine the influence of nonunion on functional outcomes after initial management. Future studies will focus on analysis of the characteristics specific to those patients diagnosed with nonunion, including treatment strategies employed and ultimate rates of recovery after nonunion.

## Conclusion

Recent studies have challenged previous standards of initial nonoperative management of humeral shaft fractures in favor of early surgical intervention, specifically as these treatment strategies relate to the risk of nonunion. Our analysis demonstrated increased risk of nonunion with initial nonoperative management. Patient factors including alcoholism and hypothyroidism additionally increased risk of nonunion, in agreement with previous reports of comorbidities resulting in metabolic derangement as increasing nonunion risk. Surgical technique additionally must be considered amongst nonunion risk factors, specifically with regards to the configuration of the surgical construct in patients treated initially with ORIF. Amongst our described population, rates of multiple reoperations for patients treated surgically after nonunion diagnosis, with increased risk specifically related to post-surgical infection, highlights the relative morbidity of nonunion diagnosis in this setting. The treatment for nonunion and time to recovery after treatment are widely variable.

## Data Availability

Data will be available from corresponding author upon reasonable request.
